# Fluid responsiveness predicted by transcutaneous partial pressure of oxygen in patients with circulatory failure: a prospective study

**DOI:** 10.1186/s13613-017-0279-0

**Published:** 2017-05-23

**Authors:** Jingyuan Xu, Xiao Peng, Chun Pan, Shixia Cai, Xiwen Zhang, Ming Xue, Yi Yang, Haibo Qiu

**Affiliations:** Department of Critical Care Medicine, Nanjing Zhongda Hospital, School of Medicine, Southeast University, 87 Dingjiaqiao Rd., Nanjing, 210009 People’s Republic of China

**Keywords:** Fluid responsiveness, Transcutaneous partial pressure of oxygen, Passive leg raising

## Abstract

**Background:**

Significant effort has been devoted to defining parameters for predicting fluid responsiveness. Our goal was to study the feasibility of predicting fluid responsiveness by transcutaneous partial pressure of oxygen (PtcO_2_) in the critically ill patients.

**Methods:**

This was a single-center prospective study conducted in the intensive care unit of a tertiary care teaching hospital. Shock patients who presented with at least one clinical sign of inadequate tissue perfusion, defined as systolic blood pressure <90 mmHg or a decrease >40 mmHg in previously hypertensive patients or the need for vasopressive drugs; urine output <0.5 ml/kg/h for 2 h; tachycardia; lactate >4 mmol/l, for less than 24 h in the absence of a contraindication for fluids were eligible to participate in the study. PtcO_2_ was continuously recorded before and during a passive leg raising (PLR) test, and then before and after a 250 ml rapid saline infusion in 10 min. Fluid responsiveness is defined as a change in the stroke volume ≥10% after 250 ml of volume infusion.

**Results:**

Thirty-four patients were included, and 14 responded to volume expansion. In the responders, the mean arterial pressure, central venous pressure, cardiac output, stroke volume and PtcO_2_ increased significantly, while the heart rate decreased significantly by both PLR and volume expansion. Changes in the stroke volume induced either by PLR or volume expansion were significantly greater in responders than in non-responders. The correlation between the changes in PtcO_2_ and stroke volume induced by volume expansion was significant. Volume expansion induced an increase in the PtcO_2_ of 14% and PLR induced an increase in PtcO_2_ of 13% predicted fluid responsiveness.

**Conclusions:**

This study suggested the changes in PtcO_2_ induced by volume expansion and a PLR test predicted fluid responsiveness in critically ill patients.

*Trial registration* NCT02083757.

**Electronic supplementary material:**

The online version of this article (doi:10.1186/s13613-017-0279-0) contains supplementary material, which is available to authorized users.

## Background

The clinical determination of fluid resuscitation can be extremely difficult in critically ill patients. Although hemodynamic maximization is favored, this approach is connected to a risk of fluid overload and heart failure. Therefore, fluid optimization is proposed to benefit patients and decrease complications. As the most common approach in the clinical settings, fluid responsiveness prediction is used to evaluate fluid optimization based on the heart working on the ascending limb of the Starling curve. The gold standard of fluid responsiveness is defined as a change in the cardiac output or stroke volume ≥10–15% after rapid volume infusion of 250 ml [1].

Significant effort has been devoted to defining and developing methods for predicting fluid responsiveness, i.e., whether the patient will benefit from fluid administration. The most common and easy approach is the passive leg raising (PLR) test which transfers blood from the leg and abdominal compartments via a postural change [2, 3].

However, the parameters measured when performing the PLR test or volume expansion are invasive and time-consuming. In considering noninvasive measurements that monitor cardiovascular function and cell oxygenation might be the future of critical care [4], a completely noninvasive, atraumatic and continuous parameter to predict fluid responsiveness, especially whether the patient could benefit from fluid resuscitation, is urgently needed [5]. The transcutaneous partial pressure of oxygen (PtcO_2_), a noninvasive measure to detect tissue ischemia or inadequate perfusion [6–9], might predict fluid responsiveness. The purpose of this study was to (1) determine whether the changes in PtcO_2_ observed during volume expansion and passive leg raising can track simultaneous changes in the stroke volume, and (2) study the feasibility of predicting fluid responsiveness by PtcO_2_ during volume expansion and PLR in critically ill patients.

## Methods

### Setting

This was a single-center, prospective study conducted in the intensive care unit of a tertiary care teaching hospital. The study protocol was approved by the Ethics Committee of Zhongda Hospital, School of Medicine, Southeast University, and informed consent was given by each patient or their next of kin. Trial registration: Clinicaltrials.gov NCT02083757. Registered 07 March 2014.

### Study population

Shock patients who presented with at least one clinical sign of inadequate tissue perfusion for less than 24 h in the absence of a contraindication for fluids were eligible to participate in the study. Clinical signs of inadequate tissue perfusion were defined as systolic blood pressure <90 mmHg or a decrease >40 mmHg in previously hypertensive patients or the need for vasopressive drugs; urine output <0.5 ml/kg/h for two hours; tachycardia; lactate >4 mmol/l.

Patients who were pregnant, younger than 18 years of age, had a contraindication to the passive leg raising test, had cardiac arrhythmias, had abdominal compartment syndrome or refused to participate in the study were excluded.

### Interventions

All patients received infusions of morphine and propofol for anesthesia and sedation as well as had an arterial catheter and central venous catheter in place. Serial measurements of the heart rate, mean arterial pressure and central venous pressure were performed. Arterial and central venous gases were obtained to determine the pH, arterial partial pressure of oxygen (PaO_2_), arterial partial pressure of carbon dioxide (PaCO_2_), arterial hemoglobin saturation (SaO_2_), hemoglobin, central venous oxygen saturation (ScvO_2_) and lactate concentration. The PtcO_2_ and transcutaneous partial pressure of carbon dioxide (PtcCO_2_) were measured by TCM (TCM 4, Radiometer Copenhagen, Denmark). After an initial calibration, the sensor was placed at the chest and heated to 44 °C. Noninvasive bioreactance stroke volume monitoring was obtained using the NICOM system (Cheetah Medical, Portland, OR) with four double-electrode stickers placed on the chest wall.

The study started when patients were sedated with a constant value of the stroke volume in semi-recumbent position for 10 min and the first set of hemodynamic measurements was recorded, including the heart rate, systemic arterial pressure, diastolic arterial pressure, mean arterial pressure, central venous pressure, stroke volume, PtcO_2_ and PtcCO_2_. Then, a PLR test was performed by changing the bed from 45° head-up to a flat position with legs elevated to 45° without changing the hip angle. When PLR induced the maximal effect on the stroke volume, hemodynamic measurements were recorded again. Then, the patient was moved back to the semi-recumbent position for 20 min and another hemodynamic measurement was performed. After volume expansion by rapid infusion of 250 ml of normal saline for 10 min [10], hemodynamic measurement was performed again. During the study time, no other change in treatment was allowed.

### Statistical analysis

Fluid responsiveness is defined as a change in the stroke volume ≥10% after 250 ml of rapid saline infusion in 10 min. Patients with fluid responsiveness were defined as fluid responders, and the remaining patients were defined as fluid non-responders.

Statistical analysis of the data was performed using SPSS 16.0 (IBM, Somers, NY). Data are presented as the mean ± standard deviation (SD) if normally distributed (Kolmogorov–Smirnov test) or as median (25–75% interquartile range, IQR). Categorical variables are presented as the number and percentage. For comparison of values between different study times, the paired Student’s t test or Wilcoxon test was performed when appropriate. For the comparison between responders and non-responders, the Mann–Whitney *U* test or two-tailed Student’s *t* test was performed when appropriate. Differences were considered significant at *p* < 0.05.

Receiver operator characteristic (ROC) curves were constructed to detect the ability of PtcO_2_ to predict fluid responsiveness. When the area under the ROC curve (AUC) was greater than 0.5, the best cutoff value was defined by the value closest to the Youden index.

## Results

### Clinical characteristics of the study subjects

Thirty-four adult patients with circulatory failure for less than 24 h were included in the study. Patients were treated according to standard protocols, and no extra interventions were performed during the study. The patients’ characteristics are detailed in Table 1. The final pool of patients included 21 men and 13 women who were 71 ± 14 years old. The averaged APACHE II score was 19. Septic and hypovolemic shock are the most common types of shock. Sixty-five percent of patients were receiving norepinephrine, and 85 percent of patients were ventilated. The ICU stay was 10 (4–14) days and in-hospital mortality was 44%. Between responders and non-responders, no significant differences were found in the duration of shock prior to study inclusion, patients receiving norepinephrine and mechanical ventilation, baseline lactate, PtcO_2_ and PtcCO_2_.

**Table 1:** Characteristics of patients. *p < 0.05 versus responder

	All patients (*n* = 34)	Responder (*n* = 14)	Non-responder (*n* = 20)	*p* value
Age (mean ± SD, years)	71 ± 14	72 ± 14	71 ± 15	0.89
Gender (male/female, no. of patients)	21/13	7/7	14/6	
Patients sources				
Emergency department	12	5	7	
Surgical department	19	8	11	
Internal medicine	3	1	2	
APACHE II score	19 ± 7	19 ± 5	21 ± 7	0.53
Type of shock (no. of patients)				
Septic	22	9	13	
Hypovolemic	10	5	5	
Obstructive	1	0	1	
Cardiogenic	0	0	0	
Neurogenic	1	0	1	
Duration of shock prior to study inclusion (h)	13 ± 7	12 ± 7	14 ± 6	0.25
Patients receiving norepinephrine (no. of patients, %)	22 (65%)	10 (71%)	12 (60%)	0.53
Mechanical ventilation, *n* (%)	29 (85%)	13 (93%)	16 (80%)	0.38
Blood gas analysis				
pH	7.42 ± 0.07	7.45 ± 0.06	7.39 ± 0.07	0.19
PaO_2_ (mean ± SD, mmHg)	159 ± 43	159 ± 47	159 ± 42	0.96
PaCO_2_ (mean ± SD, mmHg)	29 ± 5	29 ± 6	29 ± 5	0.86
Hemoglobin (mean ± SD, g/l)	9.4 ± 1.7	9.2 ± 1.7	9.5 ± 1.7	0.73
FiO_2_ (%)	48 ± 7	47 ± 5	50 ± 8	0.23
ScvO_2_ (mean ± SD, %)	82 ± 6	81 ± 5	83 ± 6	0.35
Lactate (25–75% interquartile range, mmol/L)	1.0 (0.7–2.0)	0.9 (0.7–1.8)	0.9 (0.7–1.5)	0.64
PtcO_2_ (mean ± SD, mmHg)	106 ± 38	92 ± 37	114 ± 37	0.11
PtcCO_2_ (mean ± SD, mmHg)	41 ± 11	39 ± 8	42 ± 13	0.57
ICU stay (25–75% interquartile range, day)	10 (4–14)	7 (4–13)	10 (5–16)	0.75
In-hospital mortality, *n* (%)	15 (44%)	7 (50%)	8 (40%)	0.64

SD, standard deviation; APACHE II, acute physiology and chronic health evaluation II; PaO_2_, arterial oxygen partial pressure; PaCO_2_, arterial carbon dioxide partial pressure; FiO_2_, fraction of inspiration oxygen; SaO_2_, arterial hemoglobin saturation; ScvO_2_, central venous oxygen saturation; PtcO_2_, transcutaneous partial pressure of oxygen; PtcCO_2_, transcutaneous partial pressure of carbon dioxide

### Effect on transcutaneous partial pressure of oxygen (PtcO_2_)

**Fig. 1:**
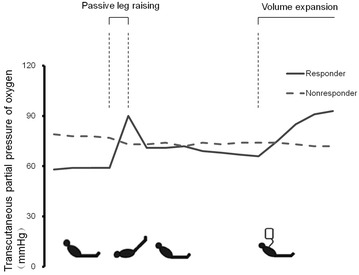
Typical recording of the transcutaneous partial pressure of oxygen before and during a PLR and then before and after volume expansion in responders and non-responders. PLR, passive leg raising

Changes in the heart rate, mean arterial pressure, central venous pressure, cardiac output, cardiac index, stroke volume, stroke volume index, PtcO_2_ and PtcCO_2_ before and during a PLR and then before and after volume expansion in responders and non-responders are displayed in Table 2. During a PLR, the mean arterial pressure, central venous pressure, cardiac output, cardiac index, stroke volume, stroke volume index and PtcO_2_ increased significantly, while the heart rate and PtcCO_2_ decreased significantly in responders. After volume expansion, the mean arterial pressure, central venous pressure, cardiac output, cardiac index, stroke volume, stroke volume index and PtcO_2_ increased significantly, while the heart rate decreased significantly in responders. However, in non-responders, the mean arterial pressure and central venous pressure increased significantly during a PLR. The mean arterial pressure, central venous pressure, cardiac output, cardiac index, stroke volume and stroke volume index increased significantly after volume expansion. Typical recording of the transcutaneous partial pressure of oxygen before and during a PLR and then before and after volume expansion in responders and non-responders is shown in Fig. 1.

**Table 2:** Hemodynamic variable at different phases in responders and non-responders during the study (*n* = 34)

Hemodynamic variable	Category	Before PLR	During PLR	Before volume expansion	After volume expansion
Heart rate (mean ± SD, beats/min)	Responder	99 ± 25	95 ± 23^a^	98 ± 25	93 ± 23^b^
	Non-responder	98 ± 24	100 ± 23	98 ± 24	97 ± 23
Mean arterial pressure (mean ± SD, mmHg)	Responder	66 ± 10	78 ± 8^a^	67 ± 10	77 ± 8^b^
	Non-responder	83 ± 13^c^	87 ± 13^a^	83 ± 12^c^	85 ± 12^b^
Central venous pressure (mean ± SD, mmHg)	Responder	8 ± 4	11 ± 5^a^	9 ± 4	11 ± 5^b^
	Non-responder	9 ± 4	11 ± 5^a^	9 ± 4	11 ± 4^b^
Cardiac output (mean ± SD, L/min)	Responder	5.5 ± 2.6	6.5 ± 2.9^a^	5.6 ± 2.5	6.5 ± 3.0^b^
	Non-responder	6.0 ± 2.3	6.1 ± 2.4	5.8 ± 2.3	6.0 ± 2.4^b^
Cardiac index (mean ± SD, l/min m^2^)	Responder	3.4 ± 1.4	4.0 ± 1.5^a^	3.4 ± 1.3	4.0 ± 1.6^b^
	Non-responder	3.4 ± 1.2	3.5 ± 1.2	3.3 ± 1.2	3.4 ± 1.3^b^
Stroke volume (mean ± SD, ml)	Responder	57.4 ± 22.7	69.5 ± 26.5^a^	58.7 ± 22.3	71.7 ± 27.1^b^
	Non-responder	63.8 ± 27.6	64.4 ± 28.7	62.6 ± 27.6	65.3 ± 30.4^b^
Stroke volume index (mean ± SD, ml/m^2^)	Responder	35.7 ± 14.0	43.4 ± 16.8^a^	36.6 ± 13.9	44.8 ± 17.0^b^
	Non-responder	36.4 ± 15.6	36.7 ± 16.1	35.8 ± 15.7	37.3 ± 17.3^b^
PtcO_2_ (mean ± SD, mmHg)	Responder	90 ± 37	101 ± 38^a^	92 ± 33	108 ± 39^b^
	Non-responder	117 ± 37^c^	116 ± 35	118 ± 37	116 ± 38
PtcCO_2_ (mean ± SD, mmHg)	Responder	39 ± 7	39 ± 7^a^	39 ± 7	39 ± 7
	Non-responder	42 ± 13	41 ± 13	41 ± 13	41 ± 12

Responders refered to patients in whom volume expansion increased stroke volume ≥10% after 250 ml of rapid saline infusion in 10 min (*n* = 14). The remaining patients were defined as non-responders (*n* = 20)

PLR, passive leg raising; PtcO_2_, transcutaneous partial pressure of oxygen; PtcCO_2_, transcutaneous partial pressure of carbon dioxide

^a^ *p* < 0.05 versus before PLR; ^b^ *p* < 0.05 versus before volume expansion; ^c^ *p* < 0.05 versus responders

**Fig. 2:**
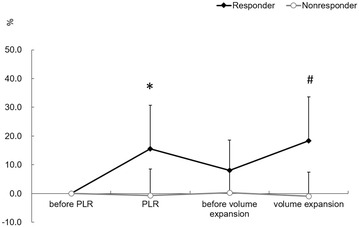
Changes in transcutaneous partial pressure of oxygen before and during a PLR and then before and after volume expansion. PLR, passive leg raising. PtcO_2_, transcutaneous partial pressure of oxygen. *p < 0.05 versus before PLR, ^#^ *p* < 0.05 versus before volume expansion

Compared with responders, the baseline mean arterial pressure and PtcO_2_ before PLR were significantly higher in non-responders, while the cardiac output and stroke volume had no significant difference from the start or during a PLR or volume expansion. Changes in the stroke volume induced either by PLR or volume expansion were significantly greater in responders than in non-responders (Fig. 2).

### Relationship between PtcO_2_ and stroke volume induced by PLR and volume expansion

The correlation between the absolute values of PtcO_2_ and stroke volume was not significant when induced by PLR or volume expansion.

Although the correlation between the changes in PtcO_2_ and stroke volume induced by PLR was not significant, the correlation between the changes in PtcO_2_ and stroke volume induced by volume expansion was significant (*r* ^2^ = 0.24, *p* = 0.004).

### Ability of the volume expansion and PLR-induced changes in PtcO_2_ to predict fluid responsiveness

**Fig. 3:**
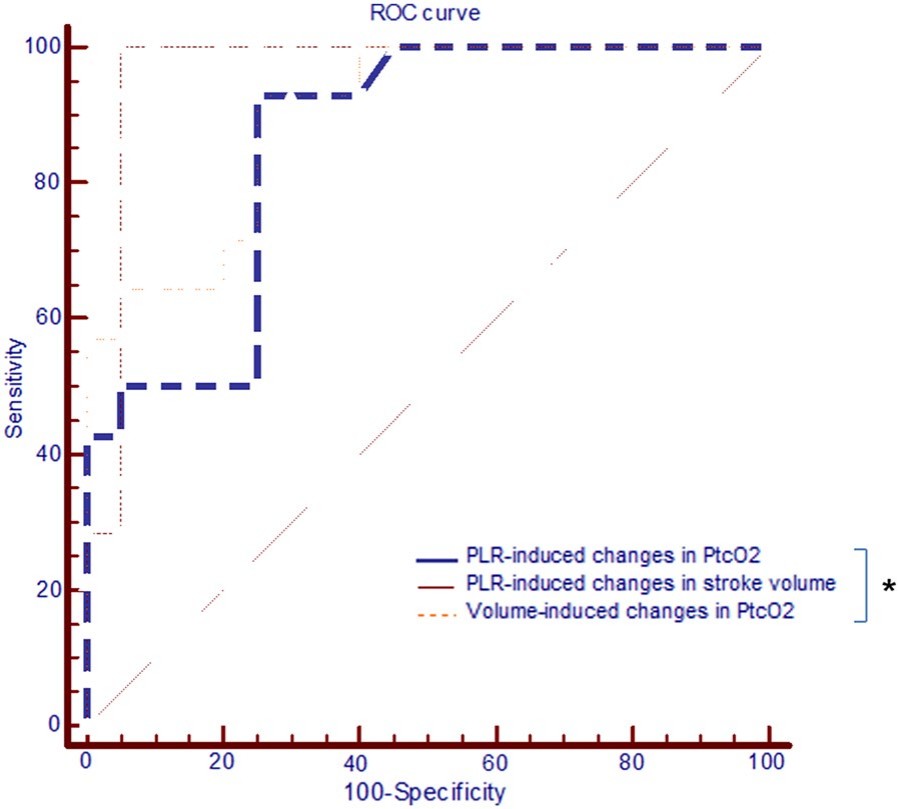
Receiver-operating characteristic (ROC) curves showing the diagnostic ability of volume expansion and PLR-induced changes in PtcO_2_ to predict volume responsiveness. PLR, passive leg raising. PtcO_2_, transcutaneous partial pressure of oxygen. *n* = 34, *p < 0.05 for the comparison between areas under the curves

A PLR-induced change in the stroke volume ≥10% predicted fluid responsiveness with a sensitivity of 100 [77–100] % and specificity of 95 [75–100] %. Volume expansion induced an increase in the PtcO_2_ of 14% predicted fluid responsiveness with a sensitivity of 93 [66–100] % and specificity of 75 [51–91] %. PLR-induced an increase in the PtcO_2_ of 13% predicted fluid responsiveness with a sensitivity of 93 [66–100] % and specificity of 75 [51–91] % (Additional file 1: Table S1; Fig. 3).

## Discussion

In this prospective study, our data suggested that PtcO_2_ could be used as a noninvasive tool that tracked the changes in stroke volume by volume expansion, and the changes in PtcO_2_ induced by volume expansion and the PLR test helped predict fluid responsiveness in critically ill patients with circulatory failure.

The aim of fluid resuscitation is to increase cardiac output and tissue perfusion, and the latter might be more important. Therefore, differentiating patients with better perfusion responsiveness to fluids matters a lot. However, the classical fluid responsiveness predicted parameters could only differentiate patients with a higher cardiac output after resuscitation (the gold standard is defined as a change in the stroke volume ≥10% after 250 ml of rapid saline infusion in 10 min). It is unknown whether tissue perfusion is improved in this scenario.

PtcO_2_ is measured by transcutaneous oxygen electrode sensors and could detect tissue ischemia or inadequate perfusion. As a continuous noninvasive parameter, in stable patients, the PtcO_2_ is a very stable variable such that even small changes are easily detectable. It has been reported that 40 mmHg for the PtcO_2_ is a critical value of death in sepsis. Tremper [11] suggested that the PtcO_2_ is an early warning sign of cardiorespiratory impairment in various shock animal models. Moreover, the PtcO_2_ is found to correlate with cardiac output. With fluid resuscitation, the PtcO_2_ responded more quickly at first than the increase in cardiac output. In a total of 1073 data sets of 106 critically ill adult patients, Tremper [12] found that the PtcO_2_ value responded quickly to changes in the blood flow with an approximate 1-min response time. Therefore, PtcO_2_ might track the change in the stroke volume, and it could predict the fluid responsiveness. It will be more meaningful if the PtcO_2_ response could replace the gold standard after fluids.

However, sometimes, cardiac fluid responsiveness may not be associated with peripheral tissue perfusion. It is reported that in septic shock patients with previous hypertension, increasing the dose of norepinephrine brings in cardiac output and microvascular improvement, but the relationship between the changes of cardiac out and microvascular variables is not related [13]. Even with increased cardiac output, the PtcO_2_ might be low, suggesting low perfusion. In our study, two patients who had a PtcO_2_ less than 40 mmHg at baseline with a relatively normal or high cardiac output did not survive.

More attention is needed when peripheral tissue perfusion is not linked to cardiac output. For example, microcirculation is responsible for regulating tissue perfusion to meet oxygen requirements. In patients with microcirculatory dysfunction, even cardiac output increased, tissue perfusion might not be changed. It is reported [14] that in delayed resuscitation situations, even fluid resuscitation enhancing cardiac output, microcirculation and tissue perfusion are not improved. Additionally, tissue oxygenation depends on adequate arterial oxygenation and total blood flow, diffusion of oxygen across capillaries and the distribution of microcirculatory flow. The capacity of tissue to take up and utilize oxygen is important as well. In shock, the PtcO_2_ might be inhibited by increased oxygen consumption of ischemic cells. Further increase in the cardiac output or oxygen no longer increases the PtcO_2_; the oxygen consumption depends on the supply, suggesting that as oxygen consumption increases, tissue hypoxia and hypoperfusion occur. Yu [7] reports that the PtcO_2_ is not changed with a higher FiO_2_ in shock, which is associated with mortality. Meanwhile, the local blood flow is always defined by local metabolic demands but not global blood flow. Therefore, even cardiac output increases, the PtcO_2_ response might not significant.

Multiple studies have demonstrated that only 40–50% of patients respond to a fluid challenge [15]. In our study, approximately 41% of patients responded to fluids, which explains why a fluid responsiveness prediction is needed before volume expansion. Moreover, simpler methods and noninvasive parameters should be used to predict fluid responsiveness.

The gold standard for assessing fluid responsiveness is defined as a change in the cardiac output or stroke volume ≥10–15% after a rapid 250 ml volume infusion [16]. However, as a simple, safe and reversible method that could promote venous blood move from the legs and splanchnic compartment to the thorax, the PLR is now used to accurately predict fluid responsiveness in most conditions [17–19]. Therefore, we focused on PLR-induced changes in parameters. In our study, the change in the stroke volume induced either by the PLR or volume expansion was significantly greater in responders than in non-responders. A PLR-induced increase in the stroke volume of 10% predicted fluid responsiveness with a good sensitivity and specificity. Volume expansion and PLR-induced change of PtcO_2_ almost had the same ability to predict fluid responsiveness. However, the correlation between the changes in PtcO_2_ and stroke volume induced by PLR was not significant. The PLR could not generate a sustained increase in the stroke volume [20] or PtcO_2_ might be the reason for this observation.

### Limitations

There are important limitations to this study. Firstly, we evaluated the stroke volume by using the NICOM system, a noninvasive bioreactance stroke volume monitoring technology [21, 22], that is not the gold standard for measuring stroke volume. However, NICOM has similar monitoring capabilities as bolus thermodilution [23, 24]. Secondly, only thirty-four patients were included in the study, and larger, higher powered trials are needed to verify the results.

## Conclusions

In this prospective study, our data suggested that the PtcO_2_ could track the changes in stroke volume, also, the changes in the PtcO_2_ induced by volume expansion and PLR test reliably predicted fluid responsiveness in critically ill patients. However, larger trials are needed to verify the results.

## Additional file

**Additional file 1: Table S1.** Feasibility of predicting fluid responsiveness by PLR-induced change in PtcO_2_

## Abbreviations

APACHE II: acute physiology and chronic health evaluation II
AUC: area under the receiver-operating characteristic curve
PaCO_2_: arterial partial pressure of carbon dioxide
PaO_2_: arterial partial pressure of oxygen
PLR: passive leg raising
PtcO_2_: transcutaneous partial pressure of oxygen
ROC: receiver operator characteristic
SaO_2_: arterial hemoglobin saturation
ScvO_2_: central venous oxygen saturation
SD: standard deviation
IQR: interquartile range
