# Supplementary material for: Fluid responsiveness predicted by transcutaneous partial pressure of oxygen in patients with circulatory failure: a prospective study
Source: Ann Intensive Care. 2017 May 23;7:56. doi: 10.1186/s13613-017-0279-0 (PMC5442033; doi:10.1186/s13613-017-0279-0)
Supplement: Supplementary file 1 — Additional file 1: Table S1. Feasibility of predicting fluid responsiveness by PLR-induced change in PtcO2 [file 13613_2017_279_MOESM1_ESM.docx]

Additional file 1: Table S1 Feasibility of predicting fluid responsiveness by PLR-induced change in PtcO_2_

| Variable | AUC | Best cutoff value | Sensitivity | Specificity | Positive predictive value | Negative predictive value | Positive likelihood ratio | Negative likelihood ratio | Youden Index |
| --- | --- | --- | --- | --- | --- | --- | --- | --- | --- |
| PLR-induced changes in stroke volume | 0.96 (0.84-0.99) | 15% | 100 (77-100) | 95 (75-100) | 93 (68-99） | 100 (82-100) | 20 (18-22) | 0 | 0.95 |
| Volume-induced changes in PtcO_2_ | 0.90 (0.75-0.98) | 14% | 93 (66-100) | 75 (51-91) | 72 (47-90) | 94 (69-100) | 4 (3-5) | 0.1 (0.0-0.7) | 0.68 |
| PLR-induced changes in PtcO_2_ | 0.86 (0.70-0.95) | 13% | 93 (66-100) | 75 (51-91) | 72 (47-90) | 94 (69-100) | 4 (3-5) | 0.1 (0.0-0.7) | 0.68 |

PLR passive leg raising, AUC area under the receiver-operating characteristic curve, PtcO_2_ transcutaneous partial pressure of oxygen. n = 34; mean (95 % confidence interval)
